# Supplementary material for: TAZ couples Hippo/Wnt signalling and insulin sensitivity through Irs1 expression
Source: Nat Commun. 2019 Jan 24;10:421. doi: 10.1038/s41467-019-08287-x (PMC6345998; doi:10.1038/s41467-019-08287-x)
Supplement: Supplementary file 1 — Supplementary Information [file 41467_2019_8287_MOESM1_ESM.pdf]

Supplementary Information

TAZ couples Hippo/Wnt signalling and insulin sensitivity through *Irs1* expression

Jun-Ha Hwang, A Rum Kim, Kyung Min Kim, Jung Il Park, Ho Taek Oh, Sung A Moon, Mi Ran Byun, Hana Jung, Hyo Kyung Kim, Michael B. Yaffe, Eun Sook Hwang, Jeong-Ho Hong

This file includes:

Supplementary Figures 1–11

Supplementary Table 1

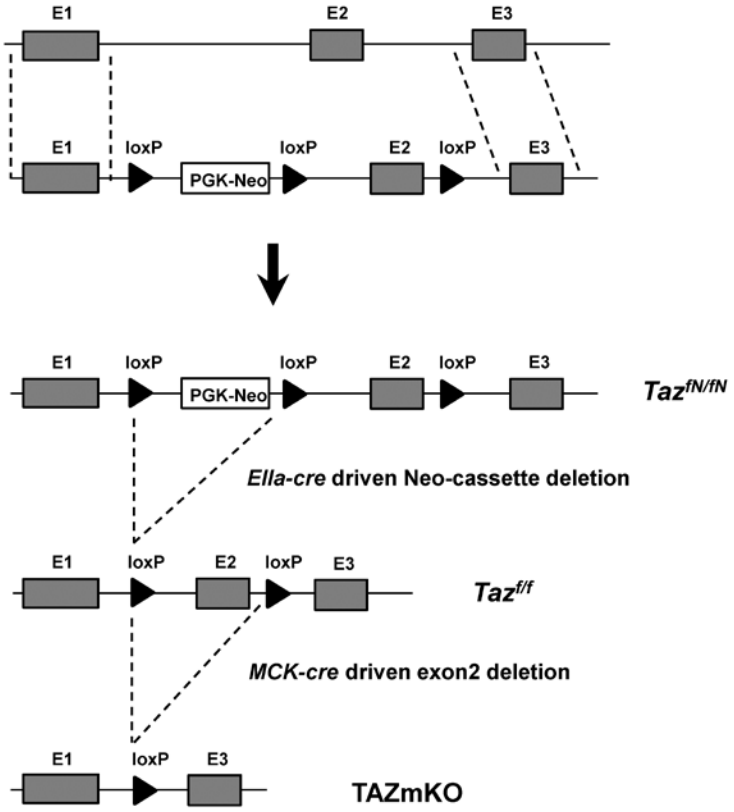

Supplementary Figure 1. Scheme depicting the generation of TAZ conditional knockout mice. The TAZ allele contains LoxP sites flanking exon 2. After deletion of the neomycin cassette, via EIIa-Cre-mediated recombination, mice were crossed with muscle creatine kinase (MCK)-Cre transgenic mice. Exon 2 of TAZ was excised by MCK promoter-driven Cre recombinase to generate muscle-specific TAZ-knockout mice (TAZmKO).

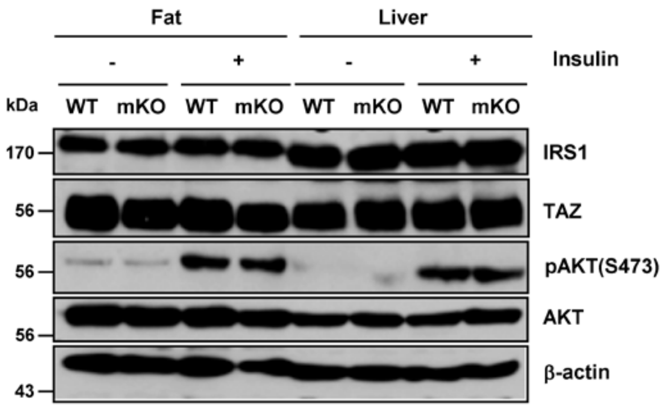

Supplementary Figure 2. Adipose and liver tissues were analysed by immunoblotting in wild-type (WT) and muscle-specific TAZ-knockout (mKO) mice. There were no differences in IRS1 expression or Akt phosphorylation after insulin injection.

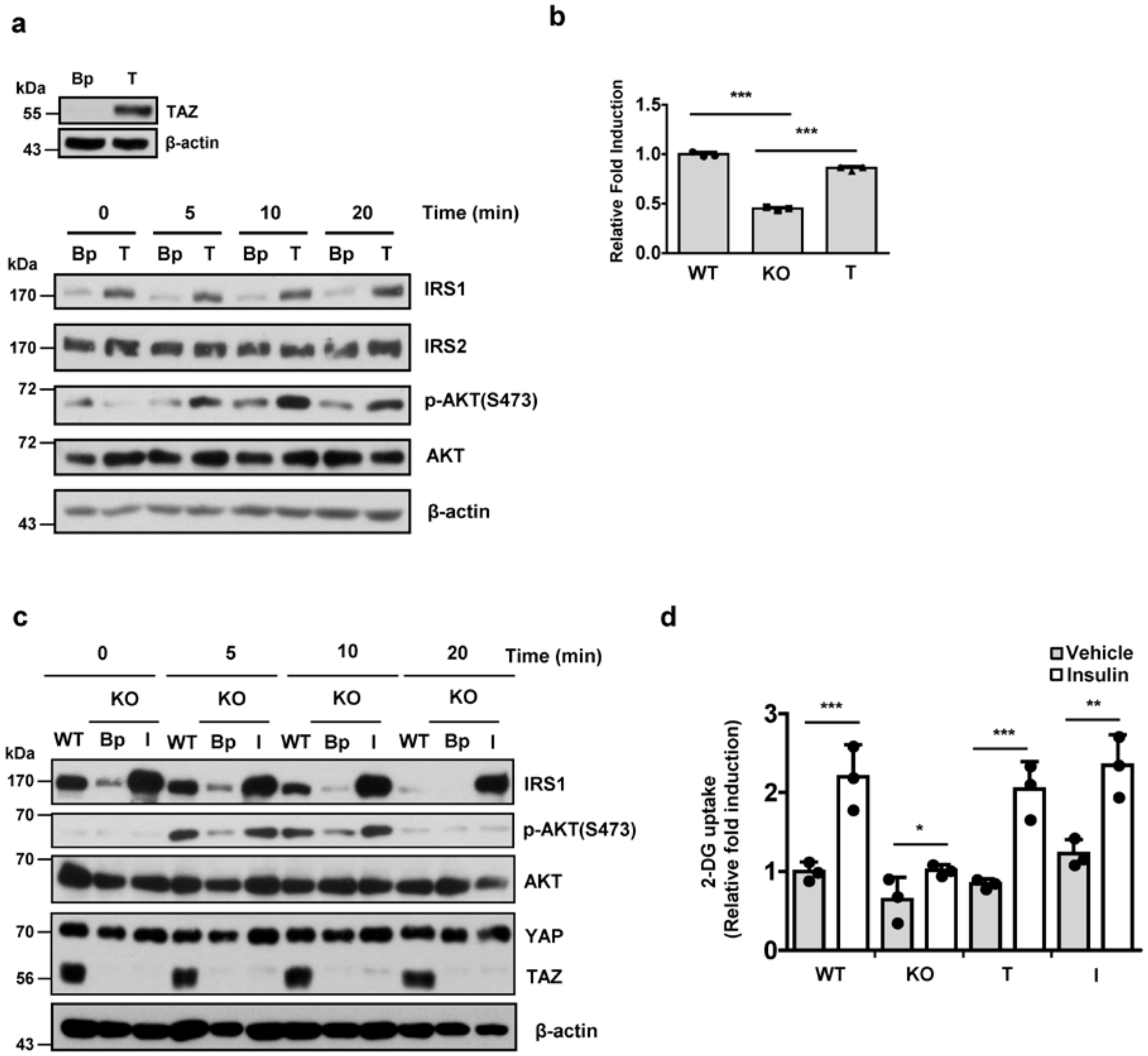

Supplementary Figure 3. (**a**) TAZ-KO MEFs were infected with control (Bp) and TAZ (T)-expressing retroviruses to establish stable cell lines (upper panel). Serum-starved Bp and T cells were treated with 1 nM insulin, and the cell lysates were used for immunoblotting. β-Actin was used as loading control. (**b**) *Irs1* expression was analysed by qRT-PCR in WT, KO, and TAZ-rescued KO (T) MEFs. (**c**) TAZ KO MEFs were transduced with control (Bp) and IRS1 (I)-expressing retroviruses to rescue *Irs1* expression. Wild type (WT), Bp, and I cells were treated with 1 nM insulin, cells were harvested, and immunoblot assay was performed. β-actin was used as the loading control. (**d**) Serum-starved WT, KO, T, and I MEFs were treated with 100 nM insulin and glucose uptake activity was analysed. Data are presented as mean ± SD values. Statistical analysis was performed using Student's t-test. * $p < 0.05$; ** $p < 0.01$; *** $p < 0.005$.

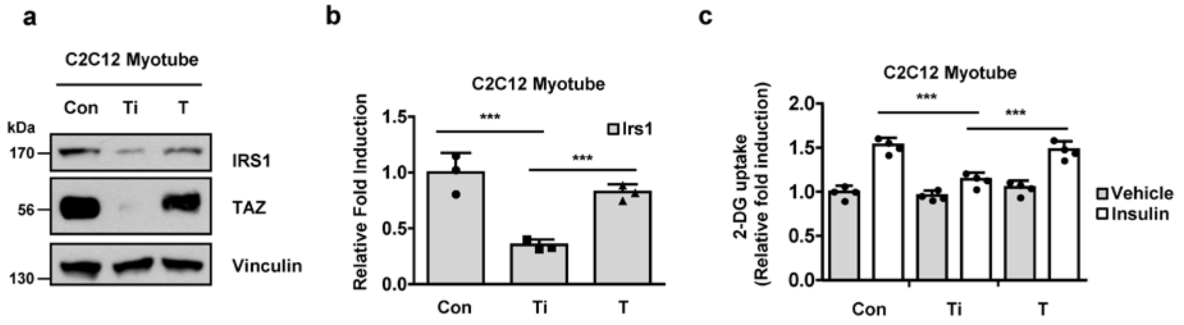

Supplementary Figure 4 (**a**) Control (Con), TAZ-knockdown (Ti), and TAZ-rescued Ti (T) C2C12 myotubes were prepared, and the level of IRS1 was analysed by immunoblotting. Immunoblot of TAZ was performed for verification of TAZ knockdown and restoration. Vinculin was used as a loading control. (**b**) Cells in panel **a** were analysed by qRT-PCR for *Irs1* transcripts (n = 3). (**c**) Serum-starved Con, Ti, and T C2C12 myotubes were treated with 100 nM insulin and glucose uptake was assessed (n = 3). For panel **b** and **c**, data are shown as mean ± SD values. Student's t-test was used for statistical analysis. *** $p < 0.005$.

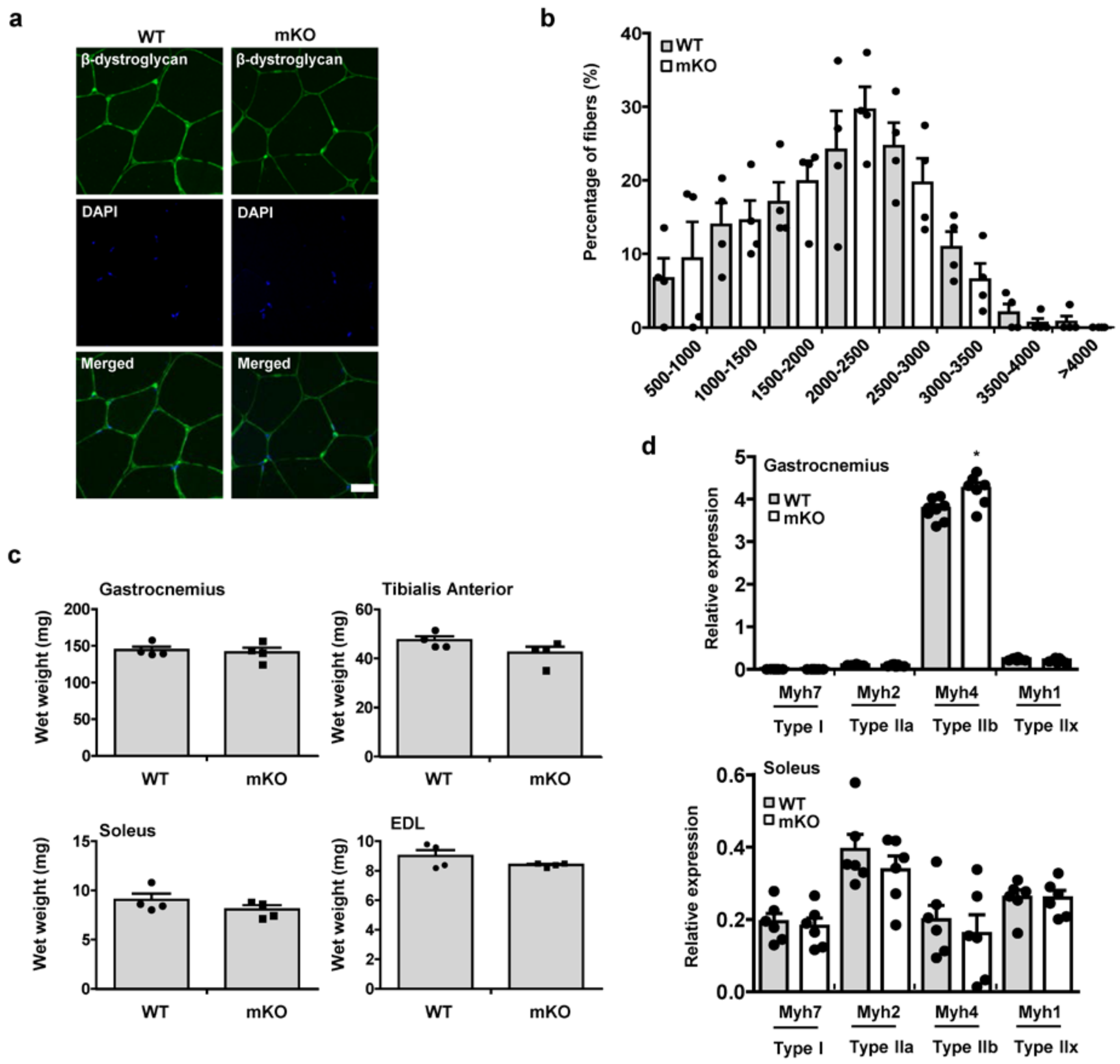

Supplementary Figure 5. (**a**) Gastrocnemius muscles of WT and TAZmKO mice were isolated, fixed with 4% paraformaldehyde, dehydrated, and embedded in paraffin. After sectioning with a microtome, specimens were stained with β-dystroglycan (green signal) to visualise the skeletal muscle fiber structure. The specimens were counterstained with DAPI (blue signal) to visualise the nuclei. Fluorescence images were obtained using a confocal microscope. (**b**) Images in panel **a** were assessed to measure muscle fibre area, using Image J software. After calculation of each fibre size, the percentage of fibres for each fibre size range is presented. Fibre size is shown as μm^2. (n = 4 for WT and mKO). (**c**) WT and TAZmKO mice were sacrificed, and the hind limb skeletal muscle was isolated immediately. The wet weights of the gastrocnemius, tibialis anterior, soleus, and extensor digitorum longus muscles were measured (n = 4). (**d**) Gastrocnemius and soleus muscle of WT and mKO mice were isolated and cDNA was synthesized from total RNA. Transcript levels of *Myh7*, *Myh2*, *Myh4*,

and *Myh1* were analysed via quantitative reverse transcriotion polymerase chain reaction to determine the composition of muscle fibre types (n = 8 for WT and n = 7 for mKO gastrocnemius muscle; n = 6 for WT and mKO soleus muscle). Data are presented as mean ± SEM values. Statistical analysis was performed using Student’s t-test. * $p < 0.05$.

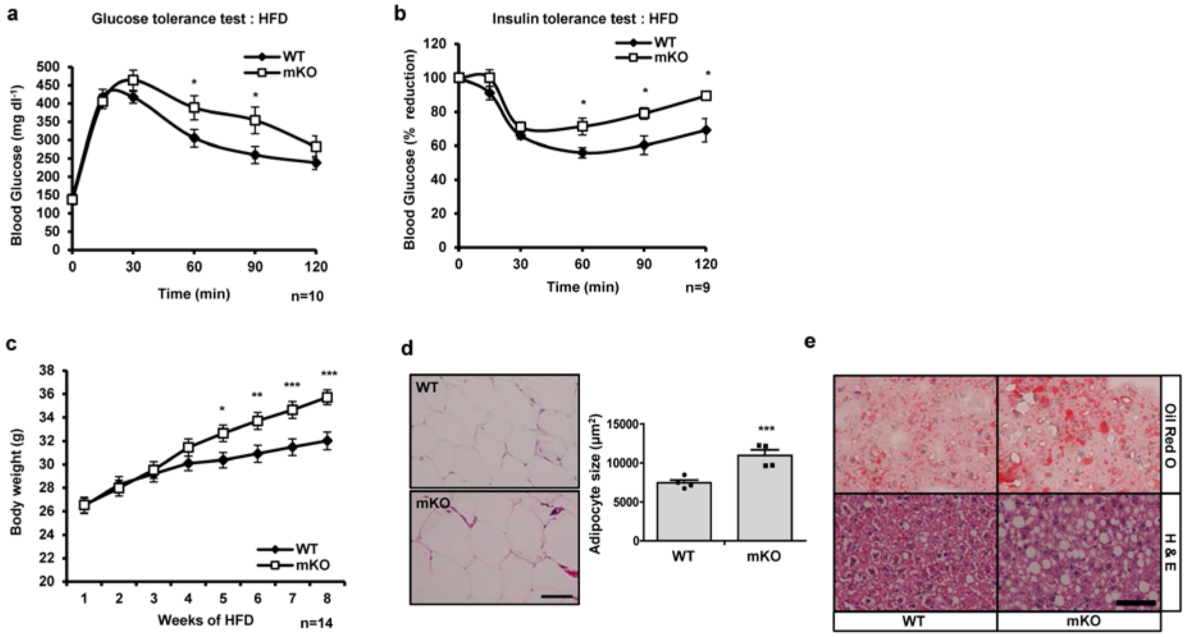

Supplementary Figure 6. (**a**) Eight-week-old WT and mKO mice were administered a high-fat diet (HFD) for eight weeks. To assess glucose tolerance, mice were starved for 16 h, and D-glucose was infused via intraperitoneal injection. Blood glucose levels were measured at the indicated time points (n = 10 for WT and mKO). (**b**) Eight-week-old WT and mKO mice were exposed to HFD condition for eight weeks and insulin tolerance was assessed. After 4 h of fasting, mice were intraperitoneally administered insulin. Blood glucose was measured at the indicated time points (n = 9 for each condition). (**c**) During 8-week HFD feeding, the body weight of mice was measured every week (n = 14 for each condition). (**d**) WT and mKO mice administered an HFD as in panel **a** were euthanised and epididymal white adipose tissue (eWAT) was isolated from HFD-fed wild type and mKO mice and fixed, dehydrated, and embedded in paraffin. Thereafter, eWAT was sectioned and analysed via hematoxylin and eosin (H&E) staining to confirm phenotypic differences between WT and mKO mice. Adipocyte size was measured using ImageJ software. n = 4 for each condition. Scale bar = 100 μm. (**e**) The liver was dissected out from HFD-fed wild type and mKO mice and fixed, dehydrated, and embedded in paraffin. After sectioning of paraffin-embedded tissue, liver

samples were analysed via H&E staining. To stain lipid droplets in the liver, isolated liver was embedded in OCT compound, frozen, and sectioned using a cryotome. Samples were stained with Oil-Red-O staining method to visualize lipid droplets in the liver. Scale bar = 100 μm. Data are presented as mean ± SEM values. Statistical analysis was performed using Student's t-test. * $p < 0.05$; ** $p < 0.01$; *** $p < 0.005$.

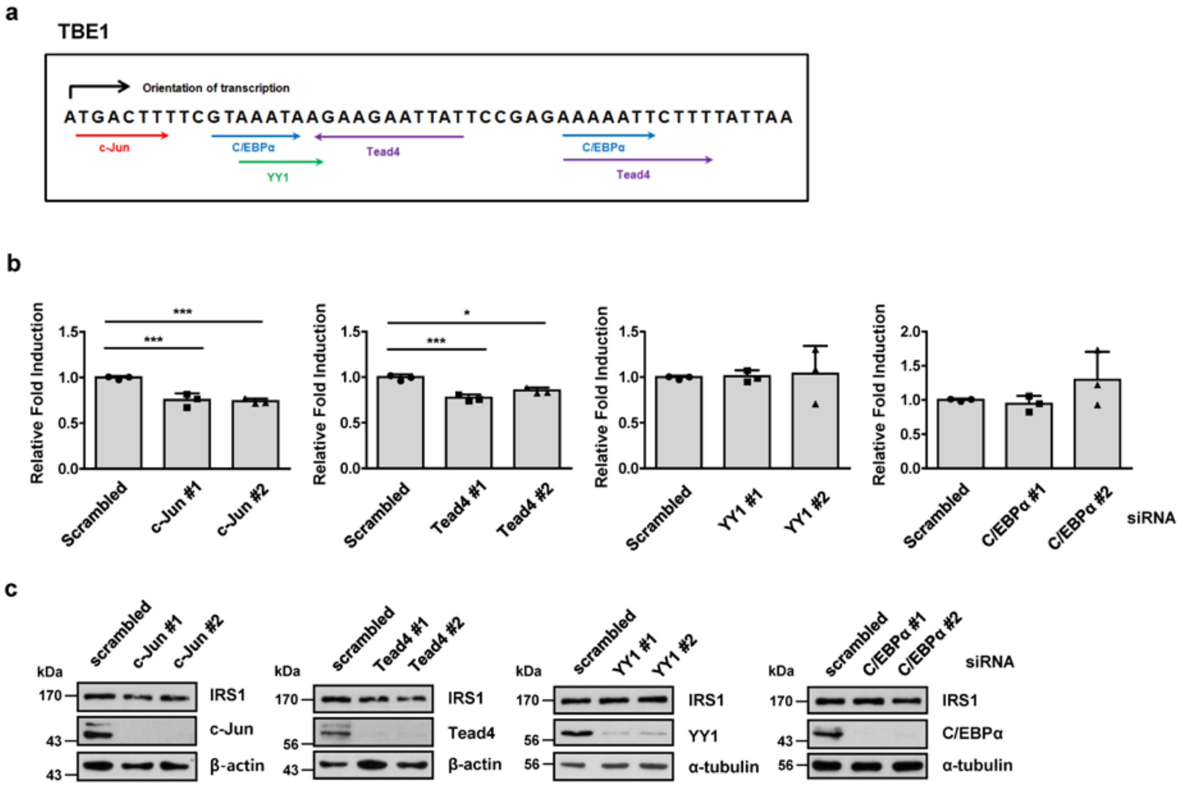

Supplementary Figure 7. (**a**) Characterization of TBE1 surrounding the *Irs1* locus. Transcription factors with the potential to interact with the TBE1 sequence were identified using programs such as PROMO and JASPAR. From the analysis, c-Jun-, C/EBPα-, YY1-, and Tead4-binding motifs were identified. (**b**) Two different siRNAs for c-Jun, Tead4, YY1, and C/EBPα were transfected into C2C12 cells. Transfected cells were analysed by qRT-PCR, 48 h after transfection (n = 3). (**c**) Cell lysates of cells described in **b** were prepared and analysed for the expression of IRS1, c-Jun, Tead4, YY1, and C/EBPα by immunoblot analysis. Data are presented as mean ± SD. * $p < 0.05$; *** $p < 0.005$.

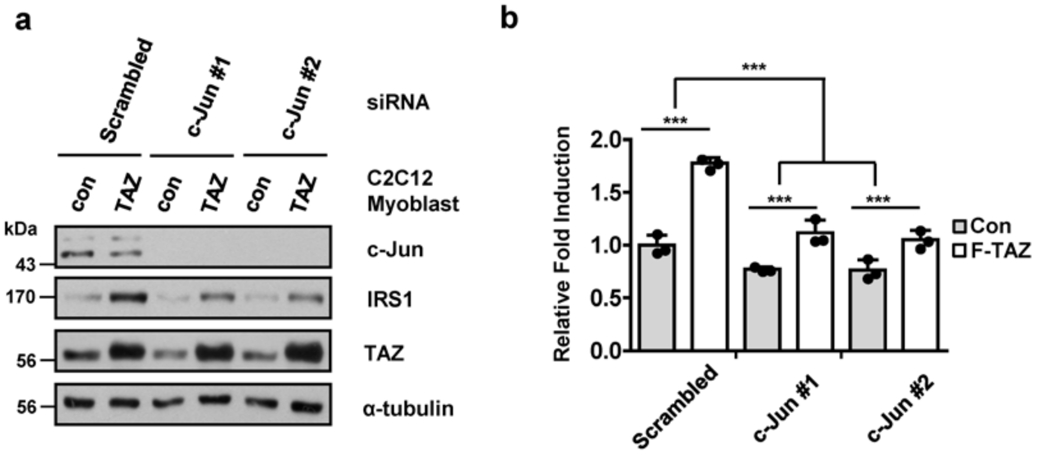

Supplementary Figure 8 (**a**) Knockdown of c-Jun abrogated F-TAZ-induced IRS1 level. Control and F-TAZ-overexpressing C2C12 myoblasts were transfected with two different siRNAs for c-Jun and analysed by immunoblotting 48 h after transfection. (**b**) Cells in panel **a** were analysed by qRT-PCR for *Irs1* transcripts (n = 3). Data are presented as mean ± SD values. Statistical analysis was performed by using student's t-test. *** $p < 0.005$.

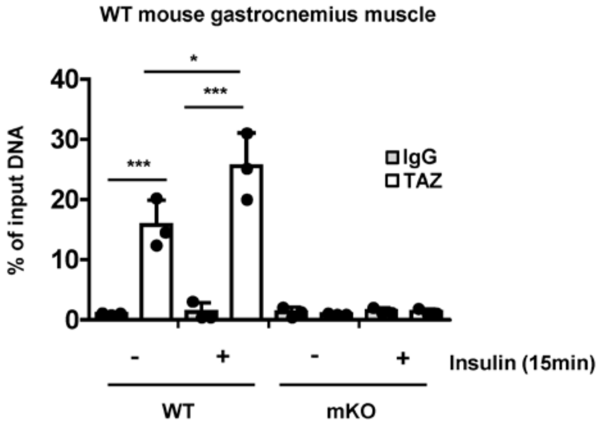

Supplementary Figure 9. WT and mKO mice were infused with insulin (1 U per kg body weight) and euthanised to dissect out the gastrocnemius muscle 15 min after infusion. After cross-linking and shearing, chromatin was prepared from muscle samples and immunoprecipitated with TAZ-specific antibody or the same amount of IgG as a control. Cross-linking of captured chromatin complexes was disrupted and released ChIPed DNA samples were purified. TAZ recruitment to *Irs1* TBE1 was assessed via quantitative polymerase chain reaction of ChIPed DNA and input DNA with TBE1 region-specific primer set. Data are shown as percentage of input DNA ± SD (n = 3 for each condition). Statistical analysis was performed using Student's t-test. * $p < 0.05$; and *** $p < 0.005$.

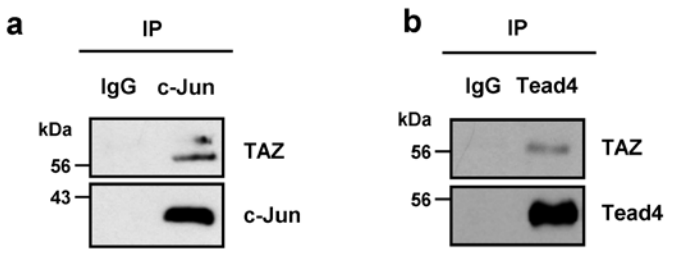

Supplementary Figure 10. (**a**) The gastrocnemius muscle of WT mice was isolated and protein lysates were prepared. Samples were immunoprecipitated with anti-c-Jun antibody and IgG as a control. After washing, protein complexes bound to beads were eluted and analysed via immunoblot assay for TAZ and c-Jun. (**b**) Protein lysates in panel **a** were immunoprecipitated with anti-Tead4 antibody and IgG as a control antibody. The eluted protein complex was assessed via immunoblotting for TAZ and Tead4.

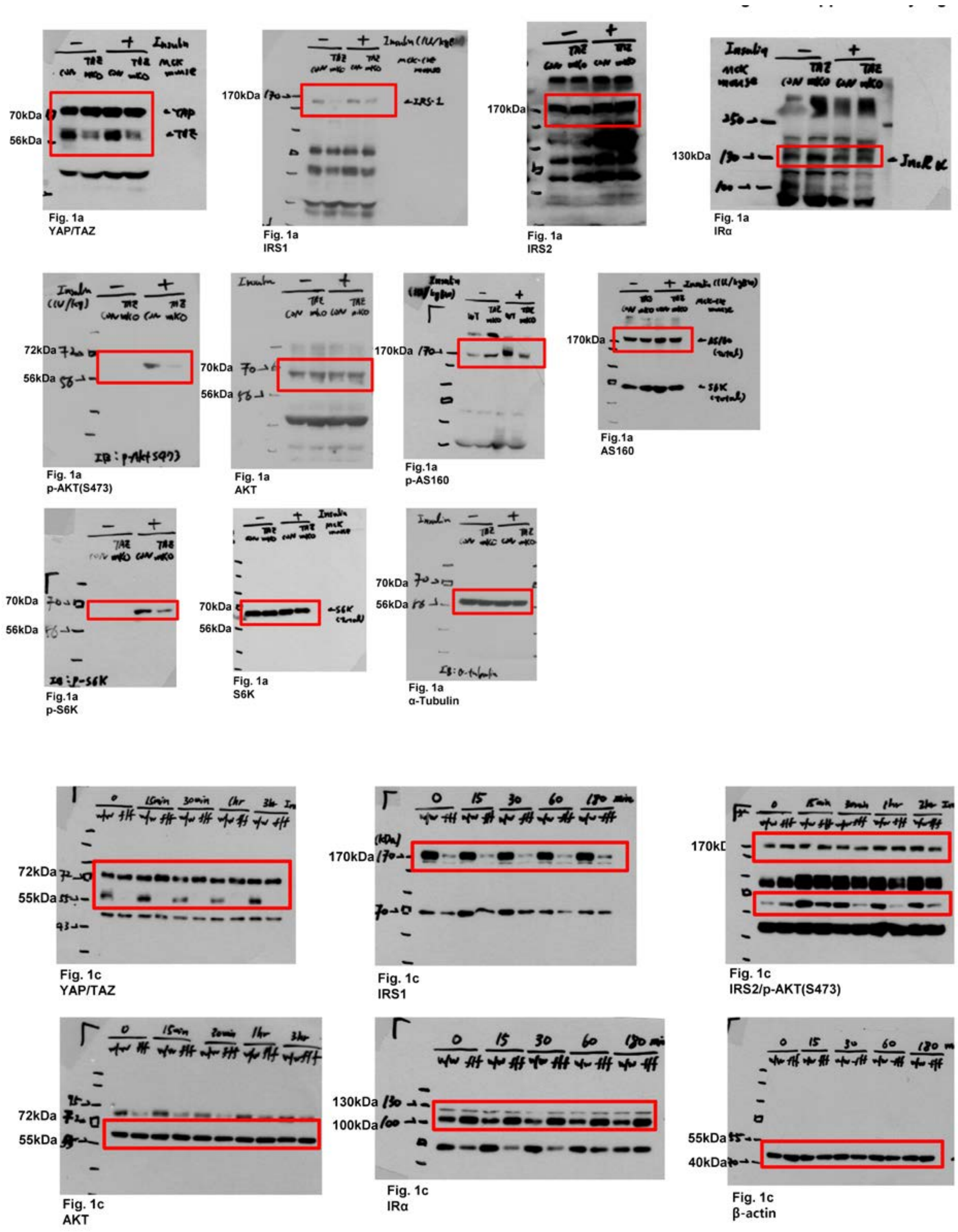

Supplementary Figure 11. Uncropped images

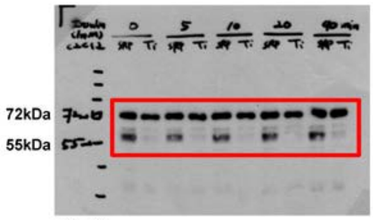

Fig. 1d
YAP/TAZ

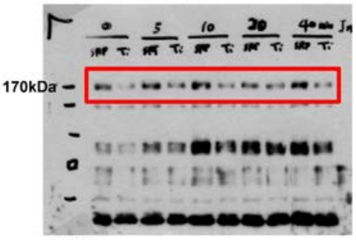

Fig. 1d
IRS1

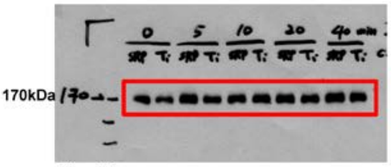

Fig. 1d
IRS2

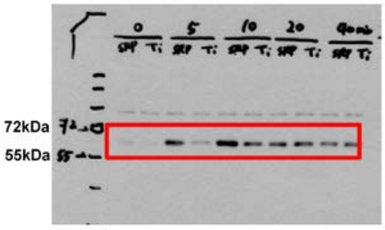

Fig. 1d
p-AKT(S473)

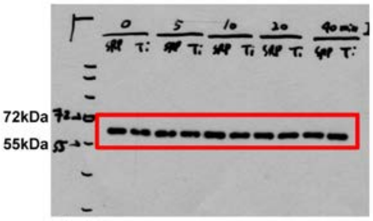

Fig. 1d
AKT

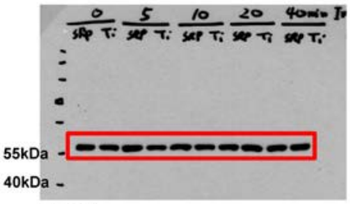

Fig. 1d
β-actin

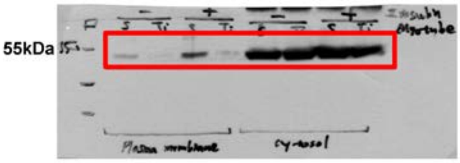

Fig. 2b
Glut4

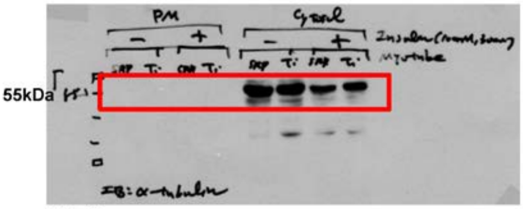

Fig. 2b
α-tubulin

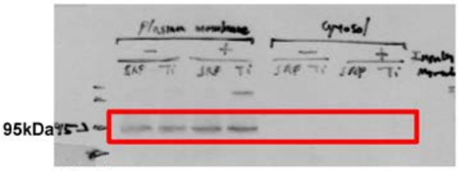

Fig. 2b
IRβ

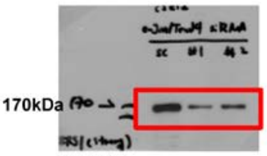

Fig. 3d
IRS1

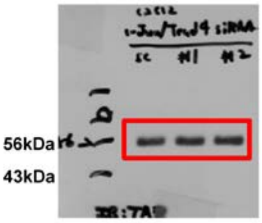

Fig. 3d
TAZ

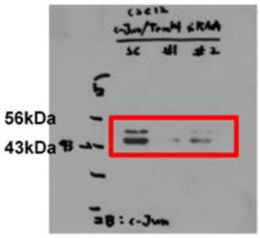

Fig. 3d
c-Jun

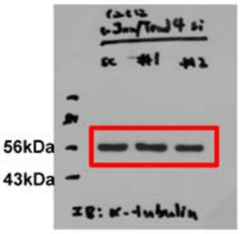

Fig. 3d
α-tubulin

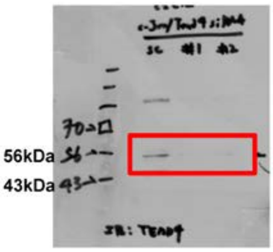

Fig. 3d
Tead4

Supplementary Figure 11. Continued

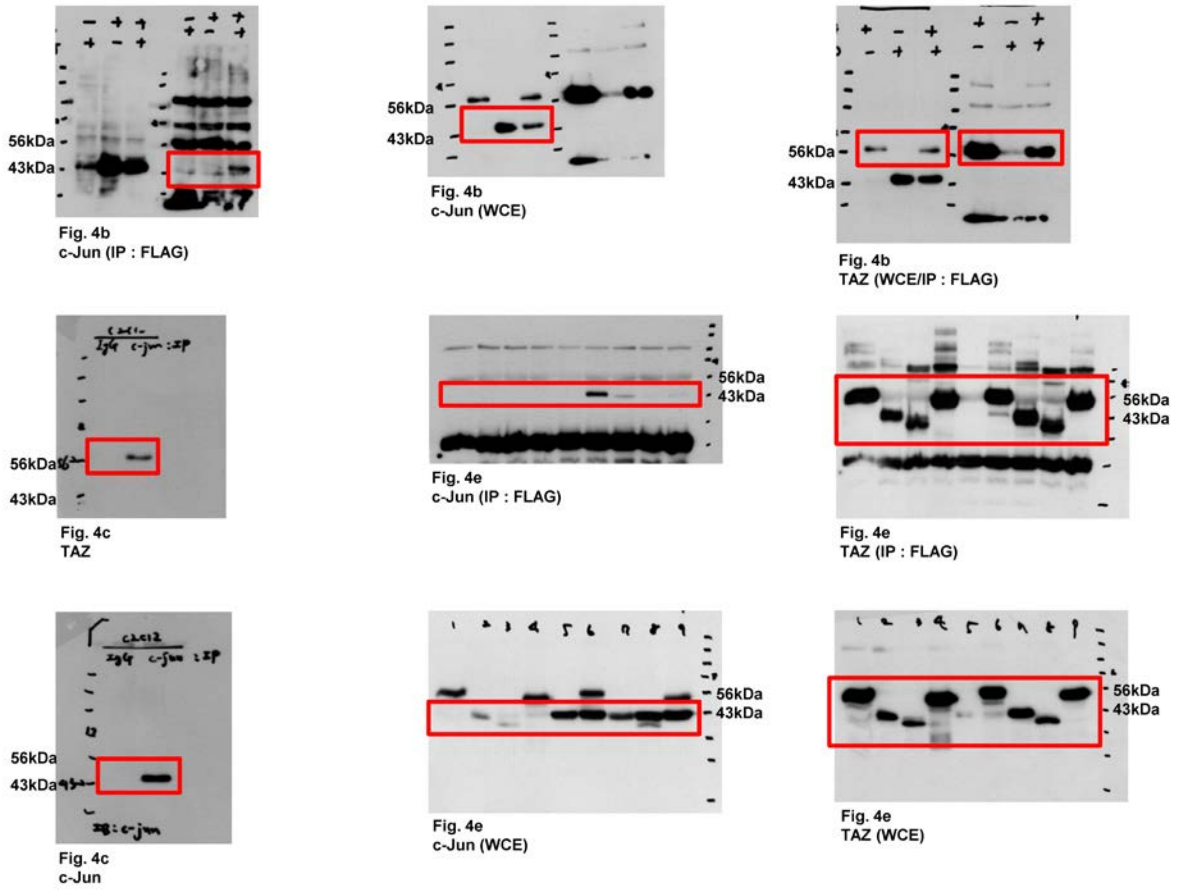

Supplementary Figure 11. Continued

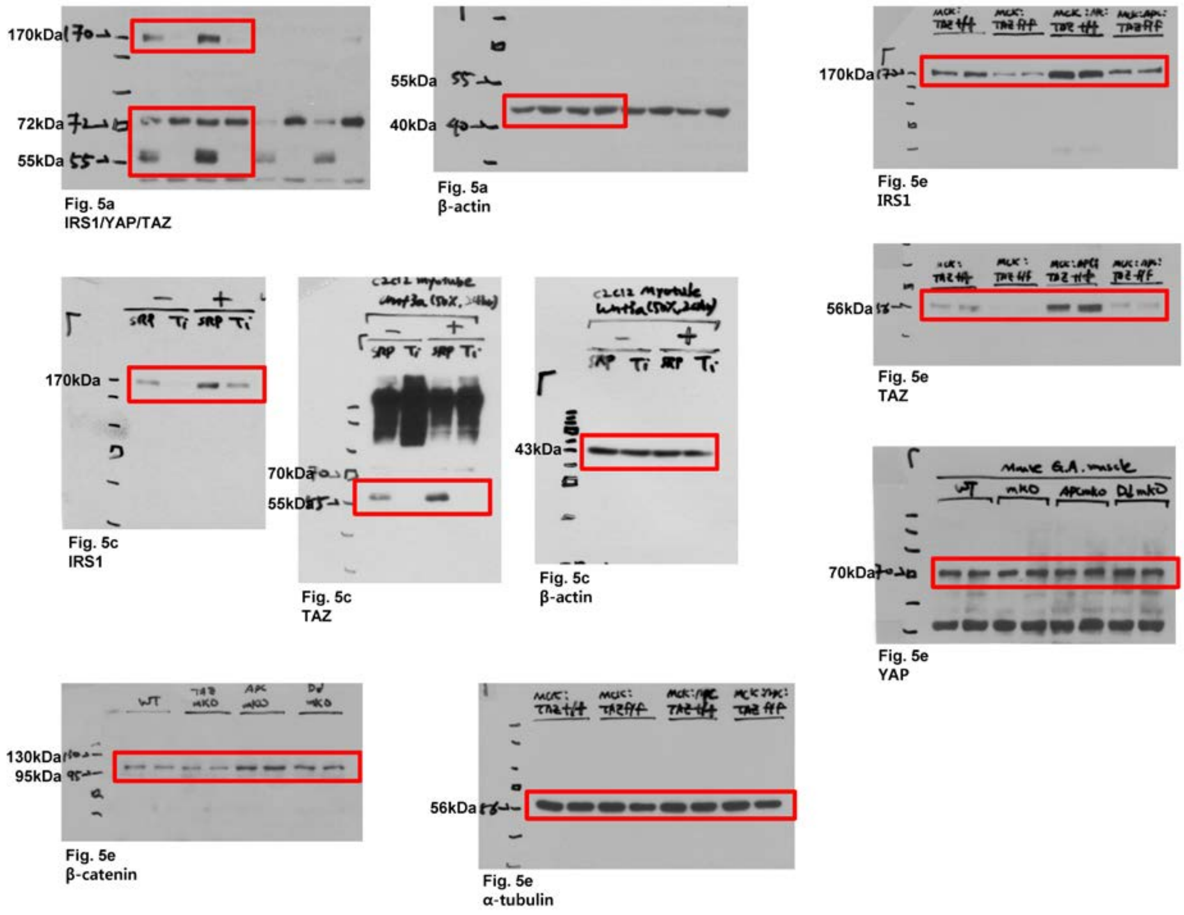

Supplementary Figure 11. Continued

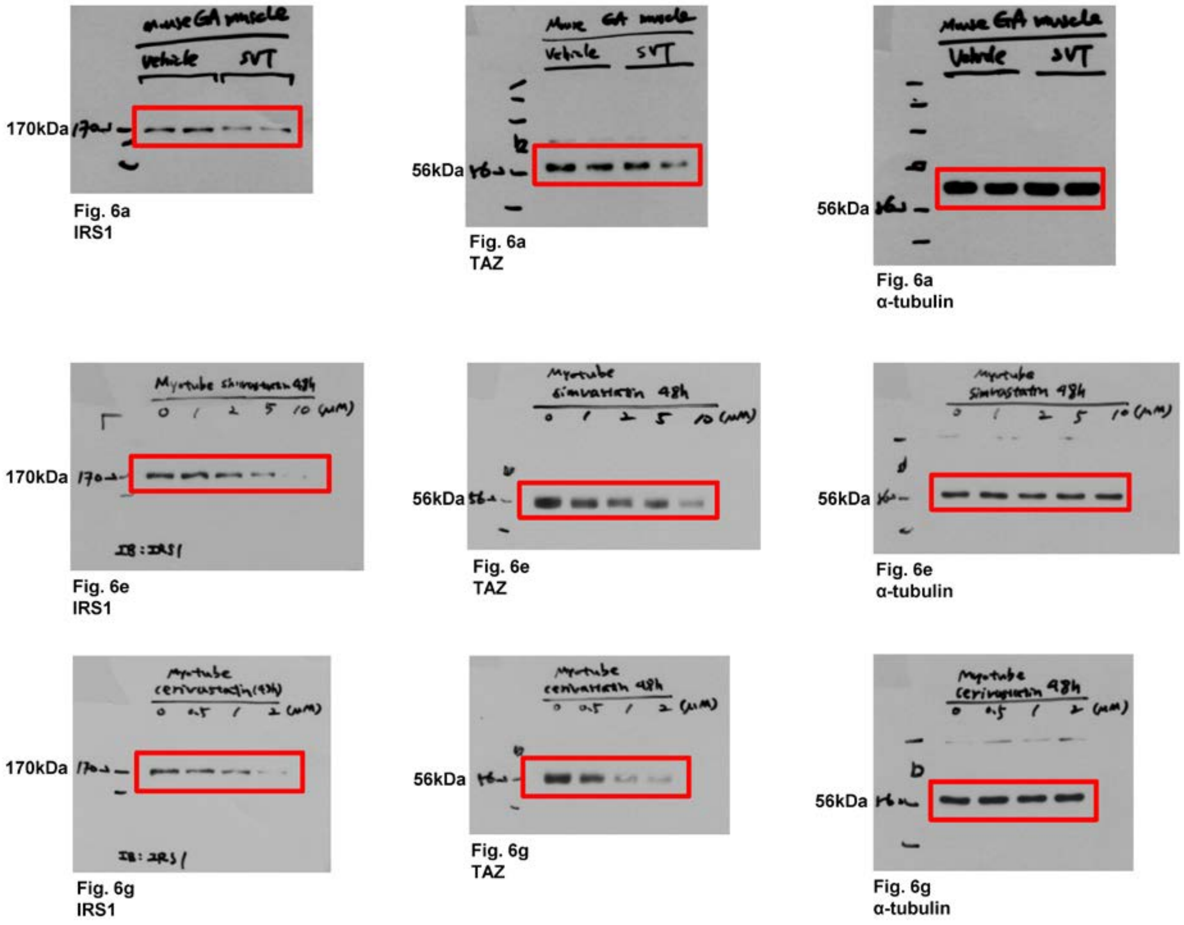

Supplementary Figure 11. Continued

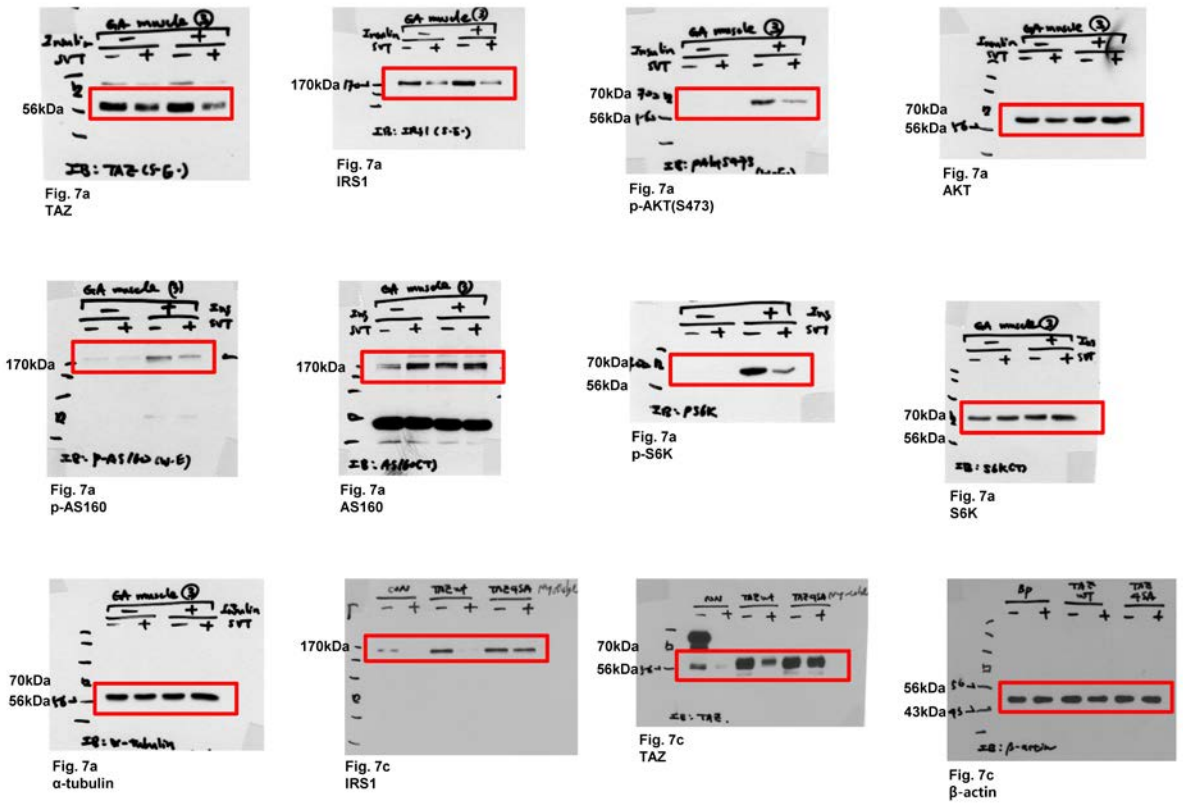

Supplementary Figure 11. Continued

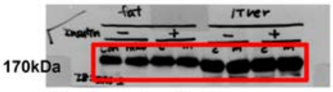

Supplementary Fig. 2
IRS1

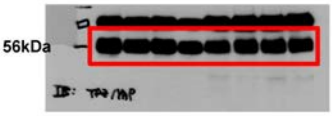

Supplementary Fig. 2
TAZ

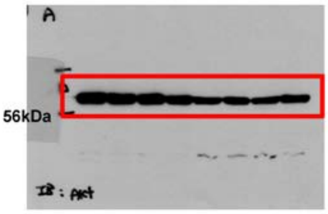

Supplementary Fig. 2
AKT

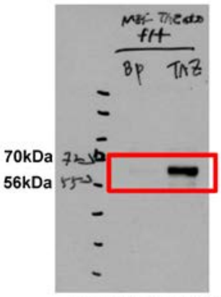

Supplementary Fig. 3a (Upper)
TAZ

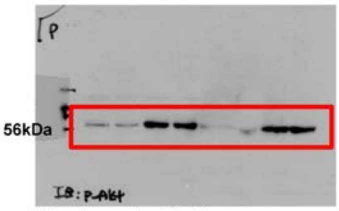

Supplementary Fig. 2
p-AKT(S473)

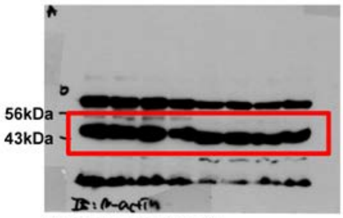

Supplementary Fig. 2
β-actin

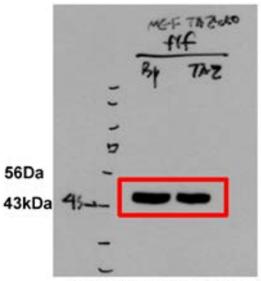

Supplementary Fig. 3a (Upper)
β-actin

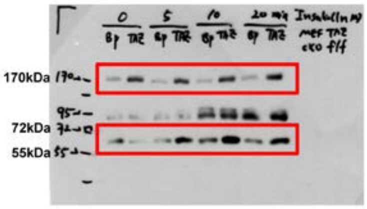

Supplementary Fig. 3a (Lower)
IRS1/p-AKT(S473)

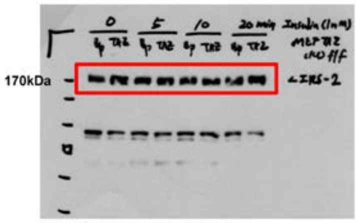

Supplementary Fig. 3a (Lower)
IRS2

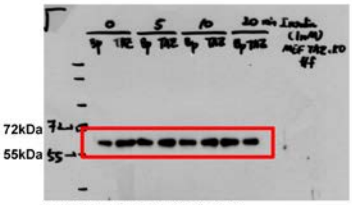

Supplementary Fig. 3a (Lower)
AKT

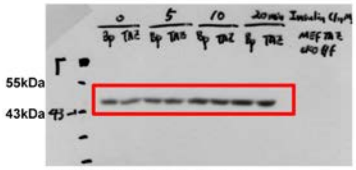

Supplementary Fig. 3a (Lower)
β-actin

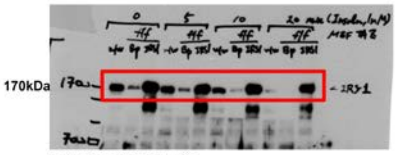

Supplementary Fig. 3c
IRS1

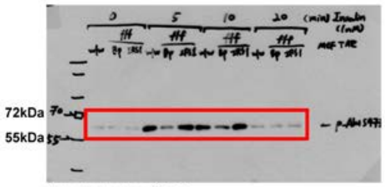

Supplementary Fig. 3c
p-AKT(S473)

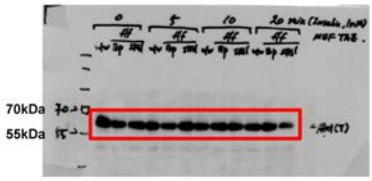

Supplementary Fig. 3c
AKT

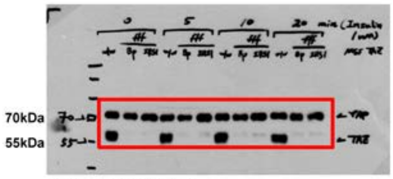

Supplementary Fig. 3c
YAP/TAZ

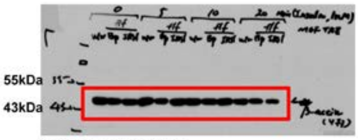

Supplementary Fig. 3c
β-actin

Supplementary Figure 11. Continued

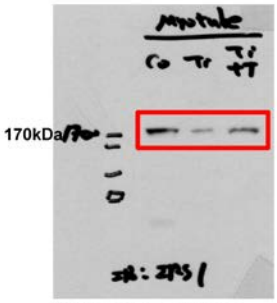

Supplementary Fig. 4a
IRS1

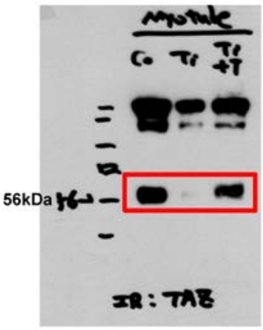

Supplementary Fig. 4a
TAZ

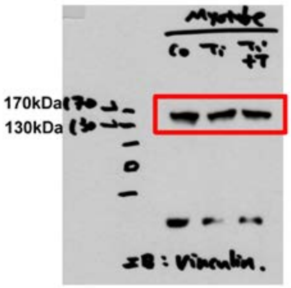

Supplementary Fig. 4a
Vinculin

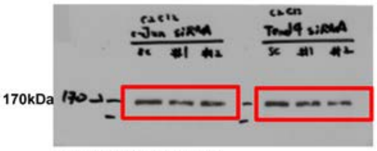

Supplementary Fig. 7c
IRS1

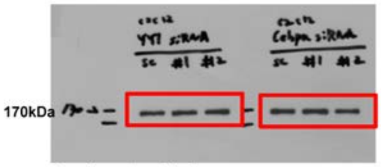

Supplementary Fig.7c
IRS1

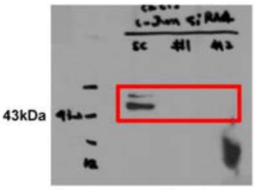

Supplementary Fig. 7c
c-Jun

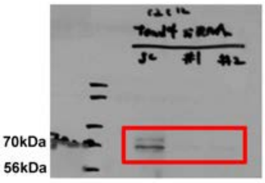

Supplementary Fig. 7c
Tead4

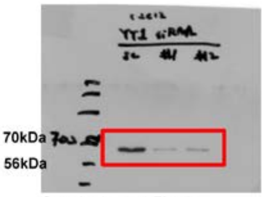

Supplementary Fig. 7c
YY1

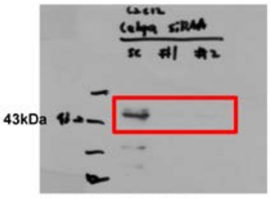

Supplementary Fig. 7c
C/EBPα

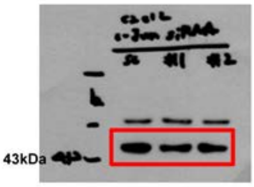

Supplementary Fig. 7c
β-actin

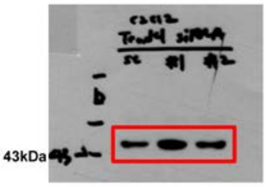

Supplementary Fig. 7c
β-actin

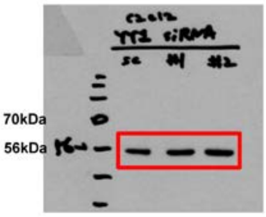

Supplementary Fig. 7c
α-tubulin

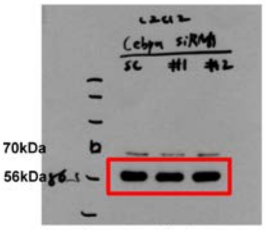

Supplementary Fig. 7c
α-tubulin

Supplementary Figure 11. Continued

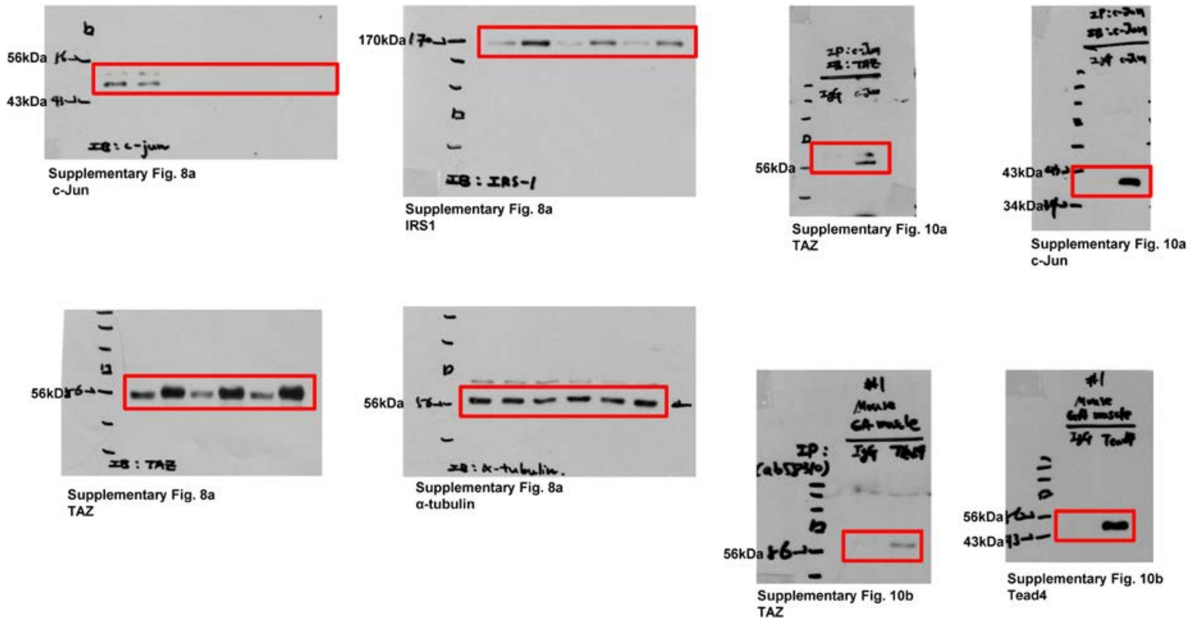

Supplementary Figure 11.

Supplementary Table 1. Used primer sequences

Primers for gene expression analysis		
gene	Direction	sequences
Irs1	forward	5'-AGCCCAAAAGCCCAGGAGAATA-3'
	reverse	5'-TTCCGAGCCAGTCTCTTCTCTA-3'
Irs2	forward	5'-AGTAAACGGAGGTGGCTACA-3'
	reverse	5'-AAGCTGCTGAGAAGTCAGGT-3'
Myh7	forward	5'-CAAGCAGCAGTTGGATGAGCGACT-3'
	reverse	5'-TCCTCCAGCTCCTCGATGCGT-3'
Myh2	forward	5'-AGAGGACGACTGCAGACCGAAT-3'
	reverse	5'-GAGTGAATGCTTGCTTCCCCCTTG-3'
Myh4	forward	5'-ACGCTTGCACACAGAGTCAG-3'
	reverse	5'-CTTGGACTCTTCCTCTAGCTGCC-3'
Myh1	forward	5'-ACCAAGGAGGAGGAACAGCAGC-3'
	reverse	5'-GAATGCCTGTTTGCCCCTGGAG-3'
Gapdh	forward	5'-GCTTGTCATCAACGGGAAG-3'
	reverse	5'-GATGTTAGTGGGGTCTCG-3'
Primers for cloning of *Irs1* enhancer luciferase reporter constructs		
Irs1 enhancer TBE1	forward	5'-AAAAAAGGTACCGCTTCGTGTTCCTCCTACTTTA-3'
	reverse	5'-AAAAAAAAGCTTGCCAAGGCTGTATTGGAAATG-3'
Irs1 enhancer TBE1 mut	forward	5'-CGAAGTGTCCTCCTCATTTTTTTTCGTAAATAAGAAG-3'
	reverse	5'-CTTCTTATTTACGAAAAAAAATGAGGAGGACACTTCG-3'
ChIP-qPCR primers of *Irs1* enhancer		
Irs1 enhancer TBE1	forward	5'-AGTACTCCTCCTGTGAAGCATA-3'
	reverse	5'-AGTTAGTTCTGTGAGCCTGTTT-3'
Non-peak region	forward	5'-CTAATCACTAACTAAAGGCTCAGAGA-3'
	reverse	5'-CACAGCCACATAATTTCAATACCT-3'
Target sequences of siRNA		
c-Jun #1		5'-GCGCACGCTCCTAAACAAA-3'
c-Jun #2		5'-GCAAATCTCTTCTGCGCCT-3'
Tead4 #1		5'-ATGTGAAACCTTTCTCTCAAAACAC-3'
Tead4 #2		5'-CTGTGAGTACATGATCAACTTTATC-3'
C/EBPα #1		5'-CCTTGAGACCGAGAGACTT-3'
C/EBPα #2		5'-CCGAGATAAAGCCAAACAA-3'
YY1 #1		5'-GCTCCAAGAACAATAGCTT-3'
YY1 #2		5'-GCGTTCGTTGAGAGCTCAA-3'
