# Supplementary material for: TAZ couples Hippo/Wnt signalling and insulin sensitivity through Irs1 expression
Source: Nat Commun. 2019 Jan 24;10:421. doi: 10.1038/s41467-019-08287-x (PMC6345998; doi:10.1038/s41467-019-08287-x)
Supplement: Supplementary file 3 — Reporting Summary [file 41467_2019_8287_MOESM3_ESM.pdf]

nature research

Corresponding author(s): Jeong-Ho Hong

Reporting Summary

Nature Research wishes to improve the reproducibility of the work that we publish. This form provides structure for consistency and transparency in reporting. For further information on Nature Research policies, see Authors & Referees and the Editorial Policy Checklist.

Statistical parameters

When statistical analyses are reported, confirm that the following items are present in the relevant location (e.g. figure legend, table legend, main text, or Methods section).

n/a	Confirmed	
☐	☒	The exact sample size (*n*) for each experimental group/condition, given as a discrete number and unit of measurement
☐	☒	An indication of whether measurements were taken from distinct samples or whether the same sample was measured repeatedly
☐	☒	The statistical test(s) used AND whether they are one- or two-sided<br>*Only common tests should be described solely by name; describe more complex techniques in the Methods section.*
☐	☒	A description of all covariates tested
☐	☒	A description of any assumptions or corrections, such as tests of normality and adjustment for multiple comparisons
☐	☒	A full description of the statistics including central tendency (e.g. means) or other basic estimates (e.g. regression coefficient) AND variation (e.g. standard deviation) or associated estimates of uncertainty (e.g. confidence intervals)
☐	☒	For null hypothesis testing, the test statistic (e.g. *F*, *t*, *r*) with confidence intervals, effect sizes, degrees of freedom and *P* value noted<br>*Give P values as exact values whenever suitable.*
☒	☐	For Bayesian analysis, information on the choice of priors and Markov chain Monte Carlo settings
☒	☐	For hierarchical and complex designs, identification of the appropriate level for tests and full reporting of outcomes
☒	☐	Estimates of effect sizes (e.g. Cohen's *d*, Pearson's *r*), indicating how they were calculated
☐	☒	Clearly defined error bars<br>*State explicitly what error bars represent (e.g. SD, SE, CI)*

Our web collection on statistics for biologists may be useful.

Software and code

Policy information about availability of computer code

Data collection	Zeiss LSM 5 software (v3.2) was used to acquire immunofluorescence images.<br>Light Cycler 480 Software (v1.5.0 SP4) was used to acquire and analyse qPCR data.<br>Bio-rad's Microplate manager software (v4.0) was used to acquire absorbance data for ELISA and 2-DG uptake assay.
Data analysis	ImageJ 1.43u was used for image analysis.<br>Microsoft Excel (v16.0) and GraphPad Prism 5 (v5.01) were used for statistical analysis.<br>Zeiss LSM Image Examiner (v4,0,0,241) was used for fluorescence image processing.

For manuscripts utilizing custom algorithms or software that are central to the research but not yet described in published literature, software must be made available to editors/reviewers upon request. We strongly encourage code deposition in a community repository (e.g. GitHub). See the Nature Research guidelines for submitting code & software for further information.

Data

Policy information about availability of data

All manuscripts must include a data availability statement. This statement should provide the following information, where applicable:

- Accession codes, unique identifiers, or web links for publicly available datasets
- A list of figures that have associated raw data
- A description of any restrictions on data availability

Uncropped images of western blot data is available in supplementary figure 11.
Raw data for ChIP-sequencing can be found at https://www.ebi.ac.uk/arrayexpress/experiments/E-MTAB-6764/ with accession number of E-MTAB-6764.

Field-specific reporting

Please select the best fit for your research. If you are not sure, read the appropriate sections before making your selection.

☒ Life sciences ☐ Behavioural & social sciences ☐ Ecological, evolutionary & environmental sciences

For a reference copy of the document with all sections, see nature.com/authors/policies/ReportingSummary-flat.pdf

Life sciences study design

All studies must disclose on these points even when the disclosure is negative.

Sample size	Sample size was not predetermined by statistical methods as similar published researches. Chosen sample sizes were sufficient to acquire statistical significance between samples in all experiments. In case of mouse samples, sample size was restricted as low as possible in the spirit of 3 R's of animal experiment as far as statistical significance of differences between genotypes or treatment is consistent and unambiguous.
Data exclusions	No data exclusions
Replication	The number of experimental replicates and number of animal were described in figure legend.
Randomization	Mice were randomly allocated to experimental group. In case of cell line, randomization was not applicable.
Blinding	There was no blinding in case of analysis by measuring quantitative parameters which is unbiased. In case of histological analysis based on stained images, mouse samples were blinded during sample processing and just identified by their mouse ID numbers.

Reporting for specific materials, systems and methods

Materials & experimental systems

n/a	Involved in the study	
☒	☐	Unique biological materials
☐	☒	Antibodies
☐	☒	Eukaryotic cell lines
☒	☐	Palaeontology
☐	☒	Animals and other organisms
☒	☐	Human research participants

Methods

n/a	Involved in the study	
☐	☒	ChIP-seq
☒	☐	Flow cytometry
☒	☐	MRI-based neuroimaging

Antibodies

Antibodies used	Anti-IRS1 (#2382), anti-p-IRS1 S307 (#2381), anti-IRS2 (#4502), anti-p-Akt T308 (#9275), anti-p-P70S6K (#9234), anti-P70S6K (#2708), anti-p-AS160 (#8730), anti-AS160 (#2670), non-phospho (active) anti-β-catenin (#8814), anti-Vinculin (#13901), and anti-TAZ/YAP (#8418) antibodies were purchased from Cell Signaling Technology. Anti-Akt (sc-8312), anti-p-Akt S473 (sc-101629), anti-IRα (sc-710), anti-c-Jun (sc-74543 for immunoprecipitation and ChIP and sc-1694 for immunoblotting), anti-YY1 (sc-281), anti-C/EBPα (sc-61), and anti-α-tubulin (sc-5286) antibodies were purchased from Santa Cruz Biotechnology. Anti-Glut4 antibody (#2203-1) was purchased from Epitomics. Anti-IR β antibody (07-724) was obtained from Millipore. Anti-FLAG (F1804) and TAZ (HPA007415) antibodies were purchased from Sigma–Aldrich. Anti-Tead4 antibody (ab97460 for immunoblotting and ab58310, for immunoprecipitation) and anti-IRS1 antibody for immunohistochemistry (ab52167) were purchased from Abcam. Anti-TAZ antibody for ChIP (NB110-58359) was purchased from Novus Biologicals.

Validation	All antibodies were validated by manufacturer, by confirming western blot bands of correct size, by specific gene knockout or siRNA-mediated knockdown experiment.

Eukaryotic cell lines

Policy information about cell lines

Cell line source(s)	C2C12 and HEK293T were purchased from ATCC. Mouse embryonic fibroblasts were isolated from wild type or whole-body TAZ KO mice embryo.
Authentication	*Describe the authentication procedures for each cell line used OR declare that none of the cell lines used were authenticated.*
Mycoplasma contamination	All cell lines showed no mycoplasma contamination.
Commonly misidentified lines (See ICLAC register)	No misidentified lines in this study

Animals and other organisms

Policy information about studies involving animals; ARRIVE guidelines recommended for reporting animal research

Laboratory animals	We used C57BL/6 strain for experiments. Only male mice were used and age of mice at experiment was described in figure legend and main text. To acquire muscle-specific Taz knockout moues, Taz floxed mice were crossed with mice bearing CRE recombinase allele in control of muscle creatine kinase promoter. Animal protocols were approved by the Institutional Animal Care and Use Committee of Korea University (Approval code KUIACUC-2018-59).
Wild animals	No wild animals were used in this study.
Field-collected samples	No field-collected samples were used in this study.

ChIP-seq

Data deposition

☒ Confirm that both raw and final processed data have been deposited in a public database such as GEO.

☐ Confirm that you have deposited or provided access to graph files (e.g. BED files) for the called peaks.

Data access links *May remain private before publication.*	https://www.ebi.ac.uk/arrayexpress/experiments/E-MTAB-6764/
Files in database submission	E-MTAB-6764.idf.txt<br>E-MTAB-6764.sdrf.txt<br>E-MTAB-6764.B.sorted.bam<br>E-MTAB-6764.T.sorted.bam
Genome browser session (e.g. UCSC)	No longer applicable

Methodology

Replicates	Four replicates of control and FLAG-tagged TAZ overexpressing C2C12 myoblasts were used. Target region in this study was also verified twice by ChIP-qPCR with same experimental procedure of ChIP.
Sequencing depth	Single end read sequencing (75bp).
Antibodies	Anti-FLAG antibody (Sigma aldrich, F1804) was used.
Peak calling parameters	Peak calling over the control sample was performed by MACS(version 2) run with default p-value cutoff of 10e-5.
Data quality	Illumina NextSeq 550 System was used for quality control. > 80% bases showed Q-score higher than 30 in all replicates. 6003 peaks were identified at q <= 0.05 and 1966953 peaks were identified at p <= 0.05.
Software	Quality Control (Nextseq 550)<br>Trimming adapter (Cutadapt v1.8)<br>Mapping (BWA mem)<br>Peak search (MACS v2)<br>Annotation (HOMER package)
